# Immune-Mediated Mechanisms in Cofactor-Dependent Food Allergy and Anaphylaxis: Effect of Cofactors in Basophils and Mast Cells

**DOI:** 10.3389/fimmu.2020.623071

**Published:** 2021-02-17

**Authors:** Rosa Muñoz-Cano, Clara San Bartolome, Rocío Casas-Saucedo, Giovanna Araujo, Sonia Gelis, Maria Ruano-Zaragoza, Jordi Roca-Ferrer, Francis Palomares, Margarita Martin, Joan Bartra, Mariona Pascal

**Affiliations:** ^1^ Allergy Section, Pneumology Department, Institut Clinic Respiratori (ICR), Hospital Clinic, Barcelona, Spain; ^2^ Asma, Reacciones Adversas y Alergia (ARADyAL), Instituto de Salud Carlos III, Madrid, Spain; ^3^ Immunoalergia Respiratoria y Experimental - Institut d'Investigacions Biomediques August Pi i Sunyer (IRCE-IDIBAPS), Barcelona, Spain; ^4^ Universitat de Barcelona, Barcelona, Spain; ^5^ Immunology Department, Centre de Diagnostic Biomedic (CDB), Hospital Clínic, Barcelona, Spain; ^6^ Centro de Investigaciones Biomédicas en Red de Enfermedades Respiratorias (CIBERES), Instituto de Salud Carlos III, Madrid, Spain; ^7^ Allergy Research Group, Instituto de Investigación Biomédica de Málaga-IBIMA, Málaga, Spain; ^8^ Biochemistry Unit, University of Barcelona Faculty of Medicine and Health Sciences, Barcelona, Spain

**Keywords:** mast cell, basophil, adenosin, NSAID, cofactor, exercise, prostaglandin, food allergy

## Abstract

Cofactors may explain why in some cases food ingestion leads to anaphylaxis while in others elicits a milder reaction or tolerance. With cofactors, reactions become more severe and/or have a lower allergen threshold. Cofactors are present in up to 58% of food anaphylaxis (FAn). Exercise, NSAIDs, and alcohol are the most frequently described, although the underlying mechanisms are poorly known. Several hypotheses have suggested the influence of these cofactors on basophils and mast cells (MCs). Exercise has been suggested to enhance MC activation by increasing plasma osmolarity, redistributing blood flow, and activating adenosine and eicosanoid metabolism. NSAIDs’ cofactor effect has been related with cyclooxygenase inhibition and therefore, prostaglandin E_2_ (PGE_2_) production. Indeed, overexpression of adenosine receptor 3 (A_3_) gene has been described in NSAID-dependent FAn; A_3_ activation potentiates Fc*ϵ*RI-induced MC degranulation. Finally, alcohol has been related with an increase of histamine levels by inhibition of diamino oxidase (DAO) and also with and increase of extracellular adenosine by inhibition of its uptake. However, most of these mechanisms have limited evidence, and further studies are urgently needed. In conclusion, the study of the immune-related mechanisms involved in food allergic reactions enhanced by cofactors is of the utmost interest. This knowledge will help to design both tailored treatments and prophylactic strategies that, nowadays, are non-existent.

## Introduction

Food allergy is the main cause of anaphylaxis in children and in some series, also in adults (1). However, only some food allergic reactions end up being an anaphylaxis, ranging from very low percentages (0.4%) up to 40% of the reactions in some series (2). This disparity may be related with differences in age, food trigger, and geographic area. Interestingly, food allergy in adults usually debuts in the second-third decade of life and, in up to 50% of the cases, as an anaphylaxis (1).

The severity of an allergic reaction is unpredictable. The same individual may present reactions with different severity, even with the same food. The presence of cofactors, such as NSAIDs, exercise or alcohol, may explain this heterogeneity. Patients with cofactor-dependent reactions may have no or mild symptoms with the food alone and a more severe reaction (generalized urticaria or anaphylaxis) when associating a cofactor. Indeed, the same patient may have reactions with different cofactors or even need more than one cofactor to develop the severe reaction. Cofactors may increase the severity of the reaction or decreasing the reactivity threshold, meaning that lower doses of allergen are able to induce a more severe reaction (from two to six times depending on the series) (2–6). Cofactors are identified in up to 58% of food anaphylaxis (FAn) in some series and related with greater severity (3, 4, 7, 8), but the underlying mechanisms are poorly understood.

Their role in anaphylaxis has been more widely studied in adult patients, being not yet optimally studied in children. The high frequency of cofactor-related reactions highlights the clinical impact of recognizing and including cofactors into the routine diagnostic workup. Indeed, the understanding of the underlying mechanisms will help in developing tailored prophylactic treatments and identifying risk biomarkers. Hereby we report the main evidence reported regarding the major cofactors involved in food allergy.

## Exercise

Exercise is described in up to 10% of anaphylactic reactions (3, 4). Exercise-induced anaphylaxis is a syndrome that may occur in food allergic patients but also independently of food ingestion (9). Considering the number of published studies, for the purpose of this review we will focus on food-dependent exercise-induced anaphylaxis (FDEIA). Several mechanisms have been postulated, although the evidence supporting each of them is scarce and limited by the low number of patients evaluated and the limited quality of the studies, as stated by a recent position paper from the European Academy of Allergy and Clinical Immunology (EAACI) (9).

The increase of both gastrointestinal permeability and tissue transglutaminase activity, two of the proposed mechanisms in the gut mucosa, is splendidly reviewed elsewhere (9). Other suggested mechanisms are related with the direct effect of exercise on basophils and mast cells (MCs) by modifying the cell count and histamine release (HR), redistributing the blood flow and increasing plasma osmolarity.

### Exercise and Basophil Reactivity and Count

Acute exercise is related with the transient increase of blood circulating neutrophils, monocytes, dendritic and NK cells, although it remains unknown whether these changes may be also related with an altered immune function (10). Some *in vivo* studies have shown an increase of basophils count and HR after exercise, being more important in atopic individuals (11). In others, only an increase in HR has been demonstrated with no modification of basophil count (12). Interestingly, some authors have not found differences in HR when comparing allergic patients with controls, regardless of a significant basophil count increase in the atopic group (11). Indeed, increase in HR after *in vitro* IgE activation was only observed in highly trained athletes compared with non-trained ones, although both groups were non-atopic (13). Thus, these observations suggest that the atopic status together with the training level might be conditioning factors of HR and therefore, of exercise-induced basophil- and MC-activation. However, the interpretation and comparison of these findings are often complicated due to differences in experimental design of the studies (*i.e.*, measurement times and methods, samples types and exercise intensity/duration).

### Exercise and Blood Flow Redistribution

It is well known that during exercise, blood flow is redistributed, being diverted from the viscera to the skeletal muscle, heart, and skin (14). Mounting evidence supports that HR during exercise is part of the physiological mechanism of recovery (15). Histamine acts as a vasodilator and is involved in post-exercise hypotension and hyperemia (15, 16). Therefore, one hypothesis that may need further exploration is whether this exercise recovery system in FDEIA patients is somehow damaged and, therefore, exercise is inducing “excessive” basophil/MC activation.

Some authors (17) have hypothesized that as a consequence of the blood flow redistribution, food allergens are transported away from the gut mucosa where resident MCs tolerate them to other tissues as skin or skeletal muscles, where MCs with a different phenotype react. For this reason, FDEIA patients tolerate the food at rest but have an allergic reaction when doing exercise. This is an interesting hypothesis and biologically plausible, although there is no experimental evidence yet.

### Exercise and Plasma Osmolarity

Another effect of exercise is plasma osmolarity increase, which magnitude depends on exercise intensity and the resulting dehydration (9). Several *in vitro* studies have demonstrated that hyperosmolar environments induce MC and basophil activation. Torres-Atencio et al. (18) showed that mannitol, as a hyperosmolar stimulus, induced significant activation in MC from LAD2 cell line and healthy individuals (MC derived from CD34+ peripheral blood cells and primary lung MC). Other authors, in three patients (one FDEIA and two food allergic) and three healthy controls, showed that changes in osmolarity increase basophil activation only in FDEIA, but not in controls or food allergic patients (19). However, the *in vitro* osmolarity achieved in that study (340 and 450 mOsm) seems difficult to be reached during exercise or dehydration in physiological conditions (293–305 mOsm) (9, 20).

### Exercise and Adenosine Metabolism

Adenosine is produced under conditions of increased energy consumption such as hypoxia or stress, rapidly increasing its circulating levels (21). Adenosine induces opposite effects in MC activation depending on the binding receptor (22). Whereas A_2A_ ligation results in an increase of cAMP, and therefore, the inhibition of MC mediator release, A_2B_ enhances MC activation through PLC. Finally, A_3_ seems to be involved in the potentiation of IgE-mediated MC activation in mouse and human models (22). Like the observations in PGE_2_–EP axis, the expression profile of adenosine receptors in the cell’s surface may condition the final effect of adenosine. Gomez et al. (23) demonstrated that adenosine enhanced IgE-mediated degranulation *via* A_3_ in human lung derived MC but not in skin MC. Interestingly, lung MCs were shown to express three-fold more A_3_ mRNA than the skin ones.

Adenosine is released into the venous efflux from skeletal muscle fibers in response to muscle contraction during exercise. Accumulated evidence shows that it is partially responsible for muscle hyperemia at submaximal and maximal workloads due to its effect on A_2A_ that results in vasodilation (24). Indeed, one of the most important factors regulating exercise capacity is the vasodilatation of the exercising muscle (25). *In vivo* studies performed in chronic heart failure patients have shown that adenosine release is impaired, partially explaining the reduced exercise capacity observed in these patients (25). Interestingly, some studies have shown that trained athletes have higher adenosine baseline plasma levels when compared with recreational ones (26). A previous publication of Muñoz-Cano et al. (27) showed that cofactor-related FAn patients (NSAIDs and exercise) overexpressed A_3_ gene (ADORA3) and others related with adenosine metabolism. Interestingly, although A_3_ activation has been linked to anti-inflammatory effects in several models of inflammation (28), it has also been related with the enhancement of IgE-mediated degranulation in human MC and, thus contributing to allergic inflammation (29, 30). Therefore, we hypothesized that the adenosine released during exercise in FDEIA patients would preferably bind A_3_ with no deleterious effect in the absence of allergen. However, in the presence of allergen, adenosine would have a synergistic effect on MC activation, favoring the allergic reaction. However, further studies need to be conducted to confirm this theory.

### Exercise and Eicosanoid Metabolism

Finally, another potential underlying mechanism in FDEIA may be related with the eicosanoid metabolism. Exercise is related with an increase in serum of products from the eicosanoid metabolism, as well as, cyclooxygenase (COX)-1 and 2-derived prostanoids (TXB_2_, PGE_2_, PGD_2_,…) and lipoxygenase (5-LOX, 12-LOX, 15-LOX) as a physiological response (31).

Different models have demonstrated that PGE_2_ abrogates IgE-mediated MC activation (32–34). Particularly misoprostol, a PGE_1_ analog, has shown to suppress symptoms in wheat-dependent exercise-induced anaphylaxis and IgE-mediated histamine release in both allergic rhinitis and healthy individuals (35–37). Rastogi et al. (38) have shown that patients with hymenoptera anaphylaxis had lower baseline PGE_2_ serum levels, suggesting that PGE_2_ may protect from anaphylaxis. Conversely, a very recent publication of Muñoz-Cano et al. (39) did not find any differences in plasma PGE_2_ at baseline in a series of FAn patients. Differences in the sample type and cause of anaphylaxis may account for this discrepancy.

Although there is no data regarding PGE_2_ levels at baseline or otherwise in FDEIA, we could suggest, as a hypothesis, that these patients may have a deficient production of PGE_2_ during exercise that would be predisposing to anaphylaxis in the presence of the allergen.

## NSAIDS

Non-steroidal anti-inflammatory drugs (NSAIDs) constitute a heterogenous group of widely used drugs with analgesic, anti-pyretic, and anti-inflammatory properties. Their main mechanism of action, despite the differences in their chemical structure, depends on prostanoid (prostaglandins and thromboxane) inhibition by blocking COX activity (40). However, some NSAIDs have COX-independent effects, such as the ability to modulate several transcription factors that control the expression of genes involved in inflammation (*f.i.* nuclear factor-kappa B) or signaling pathways (MAPK or PI3k/Akt) (41).

NSAIDs, as a cofactor, are involved in up to 25% of food-induced anaphylaxis and are considered a risk factor with an odds ratio >11 (42). Several studies have shown that NSAIDs can also induce anaphylaxis in FDEIA patients despite that NSAIDs were not originally involved in previous reactions (43, 44). The underlying mechanisms of this synergistic effect are not completely understood, and two main theories have been suggested. One is related with the alteration of intestinal permeability by NSAIDs leading to an increase of allergen absorption (45) and the other suggesting a direct effect of NSAIDs on basophils and MC.

### NSAIDs and Eicosanoid Metabolism

NSAIDs have shown to induce MC activation in certain human and animal models. In NSAID exacerbated respiratory disease (N-ERD) patients, Steinke et al. showed that aspirin induced MC activation by measuring calcium influx and PGD_2_ release (46). Interestingly, it has also been demonstrated that N-ERD patients have a decreased expression of PGE_2_ receptor 2 (EP2) that may contribute to reducing PGE_2_ capacity to mediate anti-proliferative and anti-inflammatory effects (47). Indeed, N-ERD has also decreased production of PGE_2_ (48).

Matsuo et al. (49) showed that aspirin did not induce HR by itself but enhanced IgE-mediated basophil activation. Interestingly, the authors suggested that this effect was not related with a COX-dependent mechanism but with Syk phosphorylation. On the contrary, Pascal et al. (50) showed that the ability of NSAID to enhance the IgE-mediated reactions in FAn patients may be COX1-dependent. Using a model of basophil activation test, these authors demonstrated that the activation with the allergen (peach lipid transfer protein) was enhanced by aspirin. However, this effect was not observed when co-stimulating with valdecoxib (selective COX-2 inhibitor). In the same line, Wojnar et al. showed that several chemically unrelated NSAIDs (non-selective COX inhibitors) enhanced HR induced by ragweed (51). Indeed, Matsukura (43) and Aihara (52) demonstrated a potentiation of the allergic reaction with aspirin but not with nimesulide and etodolac, both preferential COX-2 inhibitors, in FDEIA patients.

Finally, several authors have demonstrated that PGE_2_ prevents MC degranulation when acting through EP_2_ and induces a pro-inflammatory response when signaling through EP_3_ (18, 32, 53). Very recently, Rastogi et al. (38) have shown that anaphylaxis in mice can be prevented by blocking PGE_2_ degradation. They also showed that MC IgE-mediated degranulation is suppressed by PGE_2_ through EP_4_ in mouse MC and through both EP_2_ and EP_4_ ligation in human skin MC. It has been suggested that the ratio of EP receptors expressed on cell’s surface may be determinant in the final effect of PGE_2_. EP_3_ is considered to mediate pro-inflammatory effects, and EP_2_ and EP_4_ have anti-inflammatory activity (32). Also very recently, Muñoz-Cano et al. (39) showed that PGE_2_ reduced IgE-mediated basophil activation in patients with FAn. Furthermore, these authors showed a decreased expression of EP_4_ (anti-inflammatory) and increased expression of EP_3_ (pro-inflammatory) receptors in basophils. However, they did not find differences among EP pattern expression when comparing FDNIA and FAn, and all patients had a ratio EP_3_/EP_4_+EP_2_ favoring a pro-inflammatory activation upon PGE_2_ ligation.

All this suggests that eicosanoid metabolism may be involved in the development of anaphylaxis in general, and therefore, anything blocking PGE_2_ production, such as NSAIDs, may facilitate the development of a severe reaction. However, if that seems to be a universal mechanism in anaphylaxis, we must wonder why not all food allergic patients require a cofactor in order to have an anaphylaxis. In this line, Pascal et al. (50) showed that the synergistic effect of NSAID was present in both NSAID-dependent (FDNIA) and -independent FAn patients, and the main difference between them was the basophil sensitivity. Thus, FAn (NSAID-independent) patients had higher (about 148-fold) basophil sensitivity, requiring further less allergen concentration to elicit 50% of basophil maximal response compared to FDNIA patients. This suggests that enough allergen concentration could elicit an anaphylaxis in the absence of a cofactor in FDNIA. A similar observation was made in FDEIA patients *in vivo*, where the increase of the amount of allergen was enough to reproduce the anaphylaxis in the absence of exercise (43, 52, 54). Some other evidence of the differences in the pathogenic mechanism in cofactor-dependent and -independent anaphylaxis was provided by Muñoz-Cano et al. (27), who reported differences at transcriptome level. Thus, altered B-cell pathways, increased markers of neutrophil activation and reactive oxygen species levels were exclusively observed in FAn patients. However, adenosine metabolism related genes were differentially expressed only in FDNIA.

Altogether, these findings suggest that (1) eicosanoid metabolism may play a role in the development of any anaphylaxis; (2) NSAID may have a universal synergistic effect in any food allergic patient; (3) the right amount of allergen may induce an anaphylaxis in FDNIA even in the absence of a cofactor; and (4) there are other yet to be confirmed mechanisms that explain the differences between NSAID-dependent and -**in**dependent FAn.

### NSAIDs and Adenosine Metabolism

Adenosine metabolism has been linked to some NSAID-exacerbated cutaneous and respiratory diseases. ADORA3 polymorphism has been identified in NSAID-exacerbated urticaria patients (55) and ADORA1 and ADORA2A in N-ERD patients (56). Cronstein et al. (57–59) showed in a series of studies with animal and human models that NSAIDs at pharmacologic concentrations increase the release of adenosine into the extracellular milieu by uncoupling oxidative phosphorylation and, therefore, increasing ATP catabolism. These authors have suggested that the anti-inflammatory effects of NSAIDs are partly COX-independent and mediated by adenosine. However, considering that the receptor expression profile in the cell’s surface may condition the final effect of adenosine, as in the PGE_2_–EP axis, the anti-inflammatory effect of adenosine in these models may be related with a particular expression pattern of the cells/mouse strains studied.

Muñoz-Cano et al. (27) observed that FDNIA patients had a unique transcriptome signature related with adenosine metabolism genes, particularly an overexpression of ADORA3 that may be having a dual effect in these patients. A_3_ agonists have also shown anti-inflammatory effects in several mouse models due to inhibition of IFN-*γ* (60, 61). Interestingly, these authors also showed that FDNIA patients had a repressed IFN-*γ* production and IFN-*γ*-regulated genes. Considering that FDNIA patients usually have no or mild reaction when exposed to the allergen alone, A3R may be exerting its protective (anti-inflammatory) effect in this scenario through IFN-*γ* repression. However, when the patient is exposed to the food allergen plus NSAID, the adenosine released by NSAIDs, *via* A_3_, would enhance the IgE-mediated reaction, resulting in a systemic reaction. Nevertheless, further studies are still needed to completely understand the specific role of adenosine metabolism in FAn.

Finally, Pouliot et al. (62) showed that adenosine up-regulates COX-2 expression, with a consequent increase of PGE_2_ production through A_2A_. These findings suggest that the inhibitory effect of A_2A_ receptor depends on COX2-PGE_2_–EP axis. The potential connection between adenosine and PGE_2_ metabolism, both apparently involved in the development of FDNIA, opens an exciting research field that must still be developed.

## Alcohol

Alcohol is one of the classic cofactors in FAn, present in up to 15% of cases in some series (2), although the evidence supporting the underlying mechanism of its effects is scarce. We propose some hypothesis based on evidence of alcohol effect on immune cells that we briefly review hereby.

Some authors have shown that alcohol modifies intestinal permeability due to local activation of MC and modification of the expression of tight junction-associated proteins by acetaldehyde, one of its metabolites (63, 64). Actually, acetaldehyde-induced MC activation is one of the suggested mechanisms involved in alcohol-induced asthma in Japanese patients. It is well known that this population has a defective alcohol catabolism (aldehyde dehydrogenase 2 decreased activity) that facilitates acetaldehyde accumulation (64). Alcohol has also been shown to increase histamine levels by inhibiting diamino oxidase (DAO), an enzyme that catabolizes histamine (65). It has also been described that alcohol induces pro-inflammatory mediator (such IL-6, IL-10, and IFN-*γ*) release and eicosanoid metabolite production, such as PGE_2_ (66). Similar to the hypothesis in FDEIA, food-dependent alcohol-induced anaphylaxis (FDAIA) patients could have a deficient production of PGE_2_. Further studies evaluating the productions of eicosanoid metabolites in these patients would shed light upon the underlying mechanism.

Finally, adenosine metabolism again, may be involved in FDAIA. Alcohol inhibits adenosine uptake, increasing its extracellular levels (67). However, this effect is only observed in acute consumption, and chronic intake does not modify adenosine transport (67). So, as suggested in FDNIA, adenosine released upon alcohol consumption may enhance the IgE-mediated reaction induced by food allergen. Conversely to the observation in FDNIA patients, no data regarding expression profile of adenosine receptors in FDAIA exists.

## Discussion

The limited knowledge about the mechanisms involved in cofactor-enhanced FAn (CEFA) makes exceedingly difficult the development of prophylactic strategies. Apparently, avoiding strategies in CEFA patients may seem straightforward. However, in those allergic to ubiquitous allergens (*f.i.* nuts) or panallergens such as lipid transfer proteins, avoiding strategies are quite complicated considering that cofactors are everyday common situations (*f.i.* physical activity).

**Figure 1:**
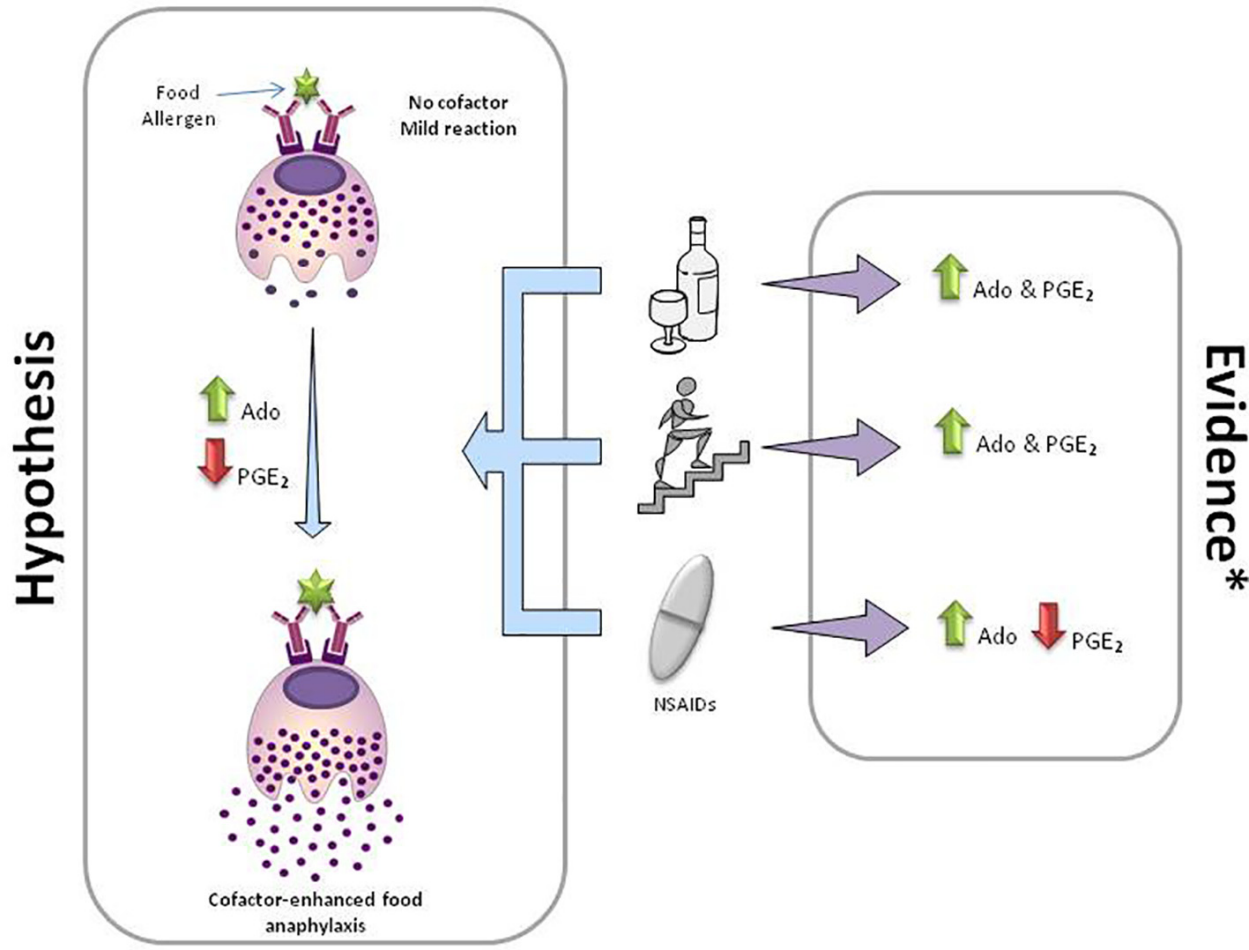
Mechanisms in cofactor-enhanced food anaphylaxis. Adenosine and eicosanoid metabolism hypothesis. The panel depicts the most frequent cofactors involved in food anaphylaxis (from top to bottom): alcohol, exercise, and non-steroidal anti-inflammatory drugs (NSAIDs). Mild reactions in the absence of a cofactor may end up in an anaphylaxis with the same amount of allergen together with the presence of a cofactor. Ado, adenosine; green arrows, increase; red arrows, decrease. *Evidence based on references (23, 26, 28, 29, 38, 39, 41, 50, 57–59, 66, 67).

Although the evidence in CEFA is limited, and we are currently working mostly based on hypothesis, the high complexity of the underlying mechanism seems evident. In the light of this data and other existent evidence not reviewed in this manuscript, several pathogenic mechanisms may be intertwined. The “cofactor effect” seems to be a universal phenomenon as demonstrated in *in vivo* and *in vitro* experiments not only in FAn patients but also in healthy individuals (43, 49, 50, 54). That means that (1) a personal predisposition may be required to develop a CEFA (or any anaphylaxis), and (2) most cofactors are interchangeable and capable of reproduce an anaphylaxis. Considering the unrelated nature of the cofactors, this observation may suggest that all of them may share, somehow, some common pathogenic mechanisms. One may suspect that all these cofactors are interfering with a compensatory system that is blocking (totally or partially) the allergic reaction induced by the food alone. The adenosine and eicosanoid metabolisms (**Figure 1**) and/or the disruption of intestinal permeability (not reviewed here) may be some of these mechanisms. In conclusion, and at risk of sounding cliché, further studies are needed to understand this “cofactor effect” and to identify risk biomarkers and prophylactic treatments.

## Author Contributions

RM-C has coordinated the different authors and written part of the manuscript. CS, RC-S, GA, MR-Z, JR-F, FP, and MM have written part of the manuscript. JB and MP have supervised the review and written part of the manuscript. All authors contributed to the article and approved the submitted version.

## Funding

RC-S is a recipient of a Rio Hortega fellowship (Carlos III Health Institute CM19/00046).

## Conflict of Interest

The authors declare that the research was conducted in the absence of any commercial or financial relationships that could be construed as a potential conflict of interest.
